# Supplementary material for: Abundance, density, and social structure of African forest elephants (Loxodonta cyclotis) in a human-modified landscape in southwestern Gabon
Source: PLoS One. 2020 Apr 29;15(4):e0231832. doi: 10.1371/journal.pone.0231832 (PMC7190099; doi:10.1371/journal.pone.0231832)
Supplement: S1 File — (PDF) [file pone.0231832.s001.pdf]

S1 File. Allelic dropout and false allele rate per locus.

Marker	Dropout	False Allele
FH126	0.032	0.00
FH48R	0.043	0.00
FH60R	0.079	0.00
FH67	0.011	0.00
FH94R	0.000	0.34
LA4	0.087	0.00
LA5	0.169	0.00
LA6R	0.180	0.00
